# Occurrence and Treatment Technologies of Per- and Polyfluoroalkyl Substances in Electroplating Wastewater: A Review

**DOI:** 10.3390/toxics14080661

**Published:** 2026-07-27

**Authors:** Yaolong Xu, Fujun Ma, Hong Chang

**Affiliations:** 1 Hebei Key Laboratory for Emerging Contaminants Control and Risk Management, College of Environmental Sciences & Engineering, Beijing Forestry University, Beijing 100083, China; 2 Institute of Soil and Groundwater Environment, Chinese Research Academy of Environmental Sciences, Beijing 100012, China; mafj@craes.org.cn

**Keywords:** PFAS, sources, occurrence, treatment technologies, target analysis, non-target screening

## Abstract

Per- and polyfluoroalkyl substances (PFASs) are a class of persistent pollutants, and China’s electroplating industry is a major source of their emissions. This paper systematically reviews the sources, occurrence characteristics, and treatment technologies of PFASs in electroplating wastewater. The chrome plating process is the primary source of PFASs, with concentrations of perfluorooctane sulfonic acid (PFOS), 6:2 fluorotelomer sulfonate (6:2 FTS), and 6:2 chlorinated polyfluoroalkyl ether sulfonate (6:2 Cl-PFESA, F-53B) in some chromium mist suppressants reaching up to 985, 735, and 875 g/kg, respectively. Monitoring data from 15 electroplating parks and 97 enterprises revealed that 29 PFAS were detected in electroplating wastewater, with long-chain compounds dominating and PFOS concentrations reaching up to 3.6 × 10^7^ ng/L. Non-target screening further identified the widespread presence of various concealed PFAS, including monohydro-substituted perfluorobutanoic acid (H-PFBA) and sodium *p*-perfluorous nonenoxybenzenesulfonate (OBS). Regarding treatment technologies, coagulation/flocculation showed very poor PFAS removal efficiency; flotation achieved up to 88% removal of perfluoroalkane sulfonic acids (PFSAs) but was constrained by process integration; biological treatment led to an increase in dissolved-phase PFAS concentrations; ion exchange resins achieved 98% removal of PFOS but were ineffective for short-chain PFASs due to their low affinity and rapid breakthrough; membrane technology achieved nearly 100% removal of long-chain PFASs but faced challenges, such as membrane fouling and concentrate disposal. This paper aims to provide a reference for PFAS pollution control in China’s electroplating industry.

## 1. Introduction

Per- and polyfluoroalkyl substances (PFASs) are synthetic persistent pollutants characterized by high-energy carbon-fluorine bonds, strong chemical and thermal stability, hydrophobicity, lipophobicity, resistance to degradation, and bioaccumulation potential [1,2,3]. Epidemiological studies have linked PFAS exposure to adverse health outcomes, including hepatotoxicity, immunotoxicity, thyroid dysfunction, reproductive and developmental effects, and increased cancer risks (e.g., kidney and testicular cancer) [4,5]. Ecological studies have also documented their bioaccumulation and biomagnification in aquatic food chains, posing risks to fish and other wildlife [6,7]. These concerns have prompted regulatory actions worldwide, including the listing of PFOS, perfluorooctanoic acid (PFOA), and perfluorohexane sulfonic acid (PFHxS) under the Stockholm Convention.

China’s electroplating industry has long been the largest in the world, with substantial chromium plating capacity and high consumption of chromium mist suppressants, making it one of the most significant industrial sources of PFAS pollution [8]. Perfluorooctane sulfonic acid (PFOS) has been widely used as a chromium mist suppressant in chromium plating. In both China and the European Union, the electroplating sector has been identified as a major contributor to total industrial PFOS emissions [9,10].

With the implementation of the Stockholm Convention, PFOS has been gradually restricted. Short-chain PFASs, 6:2 chlorinated polyfluoroalkyl ether sulfonate (6:2 Cl-PFESA, F-53B), and 6:2 fluorotelomer sulfonate (6:2 FTS) have become mainstream substitutes, increasing the complexity and diversity of PFASs in electroplating wastewater [11,12,13]. Furthermore, chemical and microbial degradation during wastewater treatment generates numerous unknown PFAS structures, and traditional target analyses may thus significantly underestimate actual pollution levels and environmental risks [14,15,16].

Most studies about PFAS removal are currently conducted under laboratory conditions, while real industrial wastewater treatment faces challenges such as complex matrices, operational fluctuations, and cost constraints [17,18]. In electroplating wastewater treatment, conventional processes like coagulation, sedimentation, and flotation generally exhibit low removal efficiencies for legacy and emerging PFASs and even lead to increased effluent concentrations due to precursor transformation or adsorption–desorption imbalances [10]. Limited field data indicate that state-of-the-art treatment technologies generally outperform conventional processes [19,20,21,22]. For example, in an electroplating park, ion exchange resin achieved 80% removal of long-chain PFOS [18]; a combined system of modified granular activated carbon and ion exchange resin increased total PFAS removal to 93–98%, but frequent adsorbent replacement raised operational costs [19].

Current reviews often focus on single technologies [18,23,24], whereas a systematic assessment of China’s electroplating industry remains lacking [25]. This review systematically summarizes PFAS sources and occurrence in electroplating wastewater, focusing on the removal patterns, mechanisms, key influencing factors, and engineering limitations of coagulation, flotation, biological treatment, adsorption, and membrane separation, aiming to provide scientific support for PFAS pollution control and process optimization in China’s electroplating industry.

## 2. Sources of PFASs in Electroplating Wastewater

**Figure 1:**
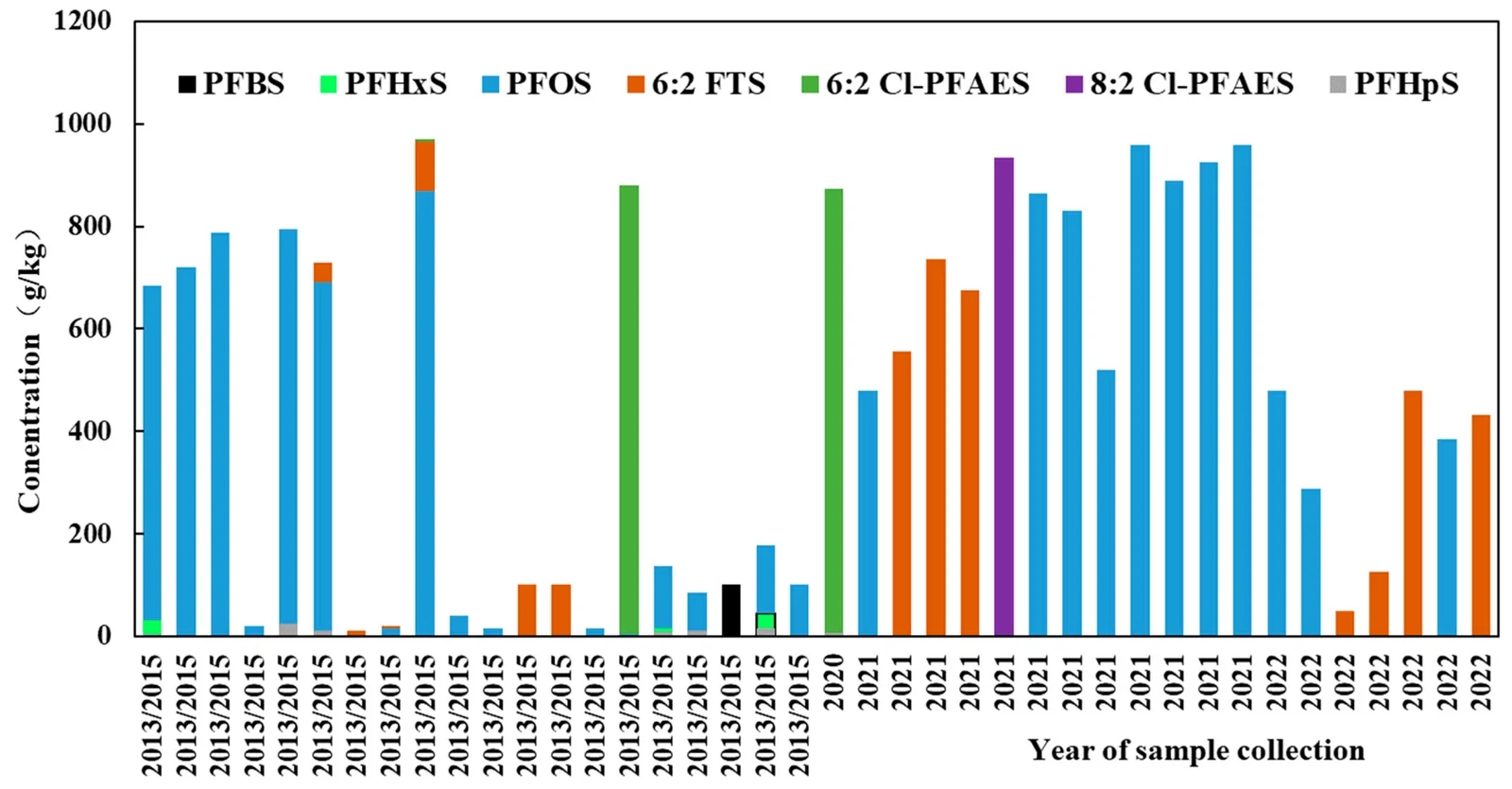
PFAS content in some chromium mist suppressants.

The electroplating industry extensively uses PFASs in chromium, nickel, copper, and zinc plating processes [15,26,27]. Among these, chromium plating is the most important source. PFOS and other PFASs are added as chromium mist suppressants to reduce electrolyte surface tension and inhibit toxic hexavalent chromium mist. According to surveys, some solid suppressants consist almost entirely of PFOS, 6:2 FTS, or 6:2 Cl-PFESA, with maximum concentrations of 958, 735, and 875 g/kg, respectively [15,20,28] (Figure 1). In non-chromium processes (e.g., pre-cleaning, nickel plating, copper plating, zinc plating), PFASs are used as bath stabilizers, wetting agents, cleaning agents, and surface additives. Unlike chromium plating wastewater dominated by PFOS, PFHxS, 6:2 FTS, and 6:2 Cl-PFESA, pre-cleaning and nickel/alkaline copper plating wastewater mainly contain PFOA, consistent with its use as a wetting and degreasing agent. PFAS concentrations in these wastewaters are typically 1–2 orders of magnitude lower than in chromium plating wastewater [19,26]. Zhang et al. [15] investigated an electroplating park in Wenzhou, confirming these differences. In 22 chromium plating wastewater samples, mean concentrations of PFOS, PFHxS, and 6:2 FTS reached 787, 140, and 122 μg/L, respectively. In contrast, pre-cleaning wastewater (*n* = 13) showed PFOA as the dominant pollutant (mean 38.5 μg/L); concentrations in nickel plating (*n* = 17), alkaline copper plating (*n* = 8), aluminum oxidation (*n* = 5), and cyanide-containing wastewater (*n* = 2) were in the single-digit μg/L range or lower.

## 3. Occurrence of PFASs in Electroplating Wastewater

Understanding the occurrence of PFASs in electroplating wastewater is essential for source identification, risk assessment, and the development of targeted treatment strategies. Based on data from 15 electroplating parks and 97 enterprises, this section summarizes the concentration levels, composition profiles, and detection frequencies of both legacy and emerging PFASs, as revealed by target and non-target analyses.

### 3.1. Target Analysis

**Figure 2:**
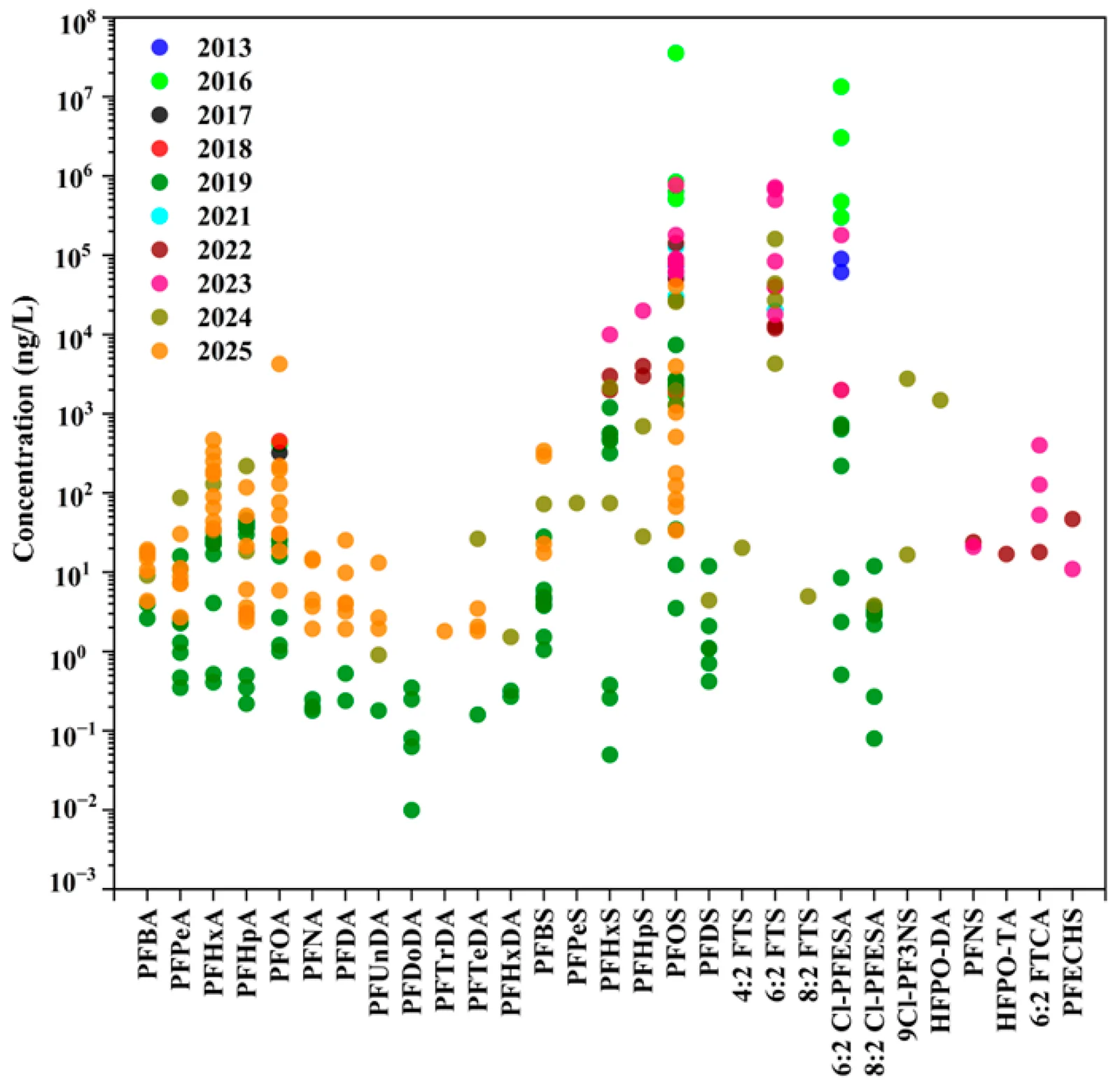
Occurrence of PFASs in electroplating wastewater based on studies published from 2013 to 2025.

Based on ten studies covering 15 electroplating parks and 97 enterprises [15,19,20,24,29,30,31,32,33], 29 PFASs were detected in electroplating wastewater, including 12 C4–C15 perfluorocarboxylic acids, 7 C4–C10 perfluoroalkane sulfonates, 6:2/8:2 Cl-PFESA, 4:2/6:2/8:2 FTS, and five emerging alternatives (hexafluoropropylene oxide dimer acid [HFPO-DA], hexafluoropropylene oxide trimer acid [HFPO-TA], 9-chloroperfluoro-3-oxanonane sulfonate [9Cl-PF3NS], 6:2 fluorotelomer carboxylic acid [6:2 FTCA], perfluoroethylcyclohexane sulfonic acid [PFECHS]), with concentrations spanning nine orders of magnitude (1.0 × 10^−2^–3.6 × 10^7^ ng/L) (Figure 2). Long-chain C8 compounds such as PFOS, 6:2 FTS, and 6:2 Cl-PFESA dominate, reflecting historical use and confirming chromium mist suppressants as the main source. PFOS was detected in 100% of samples, with a maximum concentration of 3.6 × 10^7^ ng/L and a mean of 7.8 × 10^5^ ng/L [31].

**Figure 3:**
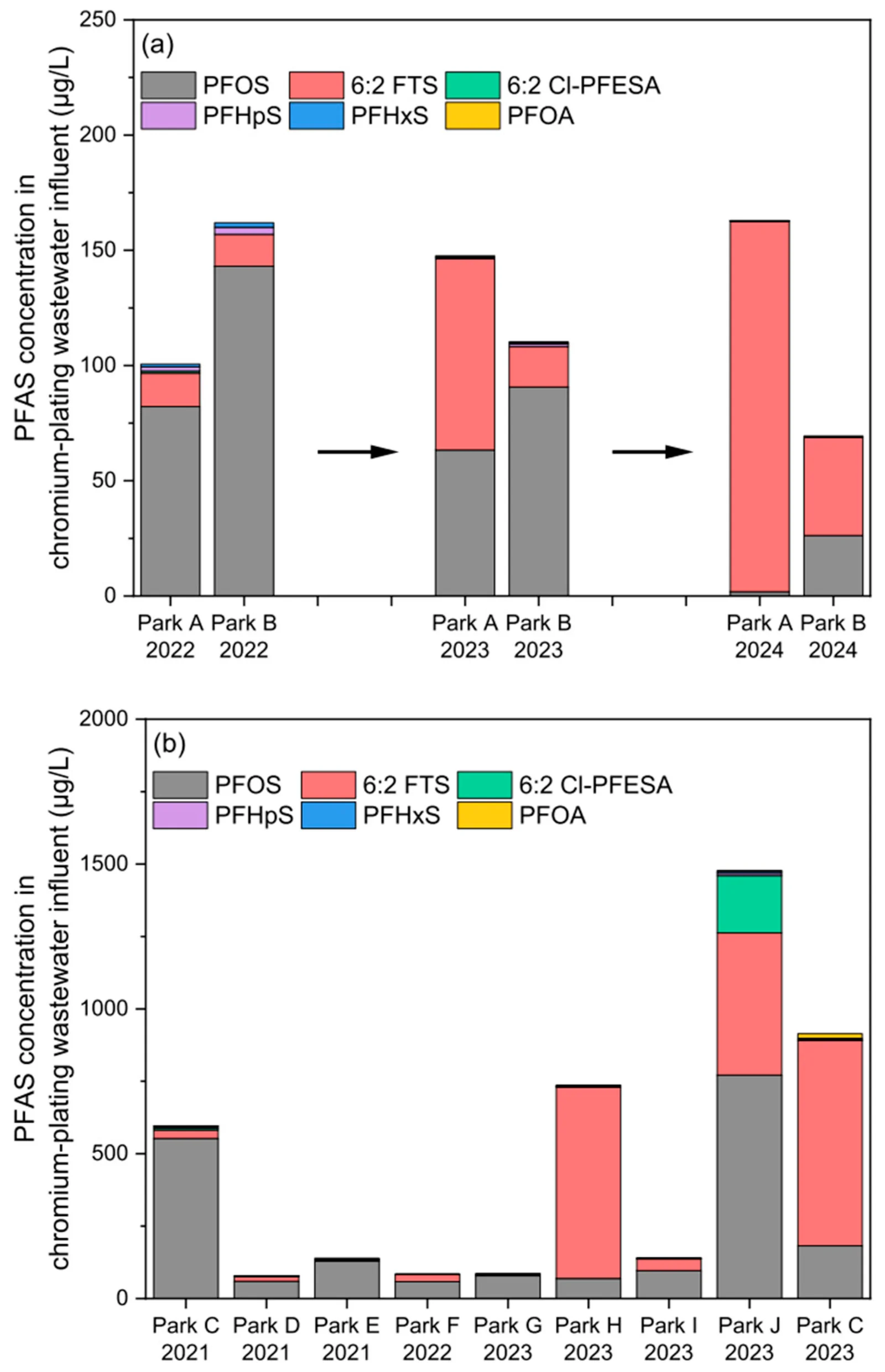
Temporal changes in PFASs in chromium-plating wastewater influent in Park A–B (**a**) and Park C–J (**b**) [15].

Data from 2013 to 2025 show that 6:2 Cl-PFESA (5.1 × 10^−1^–1.3 × 10^7^ ng/L) and 6:2 FTS (1.6 × 10^1^–7.2 × 10^5^ ng/L) are the two main PFOS substitutes. With the Stockholm Convention’s implementation, a clear temporal substitution pattern has emerged. Combined results from commercial chromium mist suppressant surveys (Figure 1) and field monitoring show that after 2022, 6:2 FTS has replaced 6:2 Cl-PFESA as the primary PFOS substitute. Jiang et al. [19] investigated 10 electroplating parks and over 50 enterprises in Guangdong and Jiangsu, indicating that China’s electroplating industry had largely transitioned from PFOS to 6:2 FTS by 2022–2024 (Figure 3). In 2022, 6:2 FTS concentrations in chromium plating wastewater were 1.2 × 10^4^–4.0 × 10^4^ ng/L, and PFOS concentrations were 5.0 × 10^4^–1.4 × 10^5^ ng/L; by 2024, the maximum 6:2 FTS concentration rose to 1.6 × 10^5^ ng/L, while PFOS concentrations generally fell below 2.6 × 10^4^ ng/L.

PFHxS (5.0 × 10^−2^–1.2 × 10^4^ ng/L) and perfluoroheptane sulfonic acid (PFHpS) (28–2.0 × 10^4^ ng/L) are frequently detected as byproducts/impurities from PFOS production and use [22]. Perfluorocarboxylic acids (PFCAs), especially PFOA, are also frequently detected, with a mean concentration (352 ng/L) approximately three orders of magnitude lower than PFOS (7.8 × 10^5^ ng/L), but still noteworthy. PFOA introduced via metal cleaners and bath additives can reach up to 4.3 × 10^3^ ng/L in some park wastewaters [33].

### 3.2. Non-Target Screening

**Figure 4:**
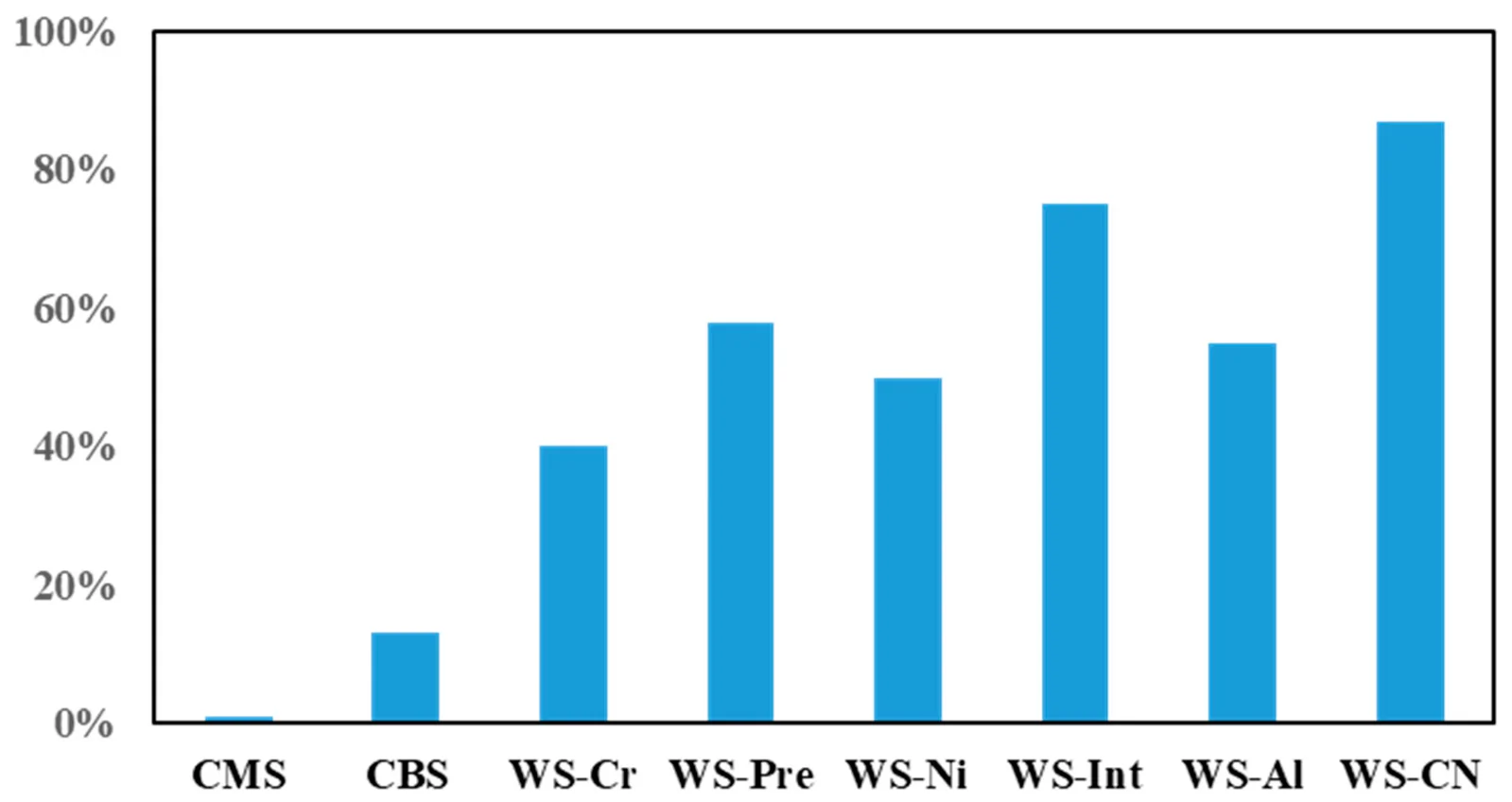
Proportions of non-target PFASs in different electroplating wastewaters [11].

The above findings are largely based on target analysis. However, with the continuous emergence of new substitutes and the transformation, degradation, and rearrangement of PFASs and precursors in complex electroplating systems, many hidden PFASs remain undetected by target methods, leading to underestimation of pollution levels and environmental risks [14,34,35]. Recent studies show that the proportion of non-target PFASs increases from 1.0% in chromium mist suppressants to 13.0% in plating baths and further to 40.0% in chromium plating wastewater [33]. In pre-cleaning, nickel plating, alkaline copper plating, aluminum oxidation, and cyanide-containing wastewater, non-target proportions are even higher: 58%, 50%, 75%, 55%, and 87%, respectively (Figure 4). Among non-target emerging PFASs, H-PFBA was detected in 71–100% of samples, reaching up to 1310 μg/L in pre-cleaning wastewater, and co-occurred with PFOA, suggesting similar sources. Monohydro-substituted perfluoroalkyl carboxylic acids (H-PFCAs) were previously found mainly in fluorochemical wastewater; their wide presence in electroplating processes may result from in situ degradation of fluoropolymers used in pre-treatment and nickel plating under strong acid, high temperature, and high current conditions [34]. Another non-target emerging PFAS, OBS, was detected across all sample types, with the highest concentrations in chromium plating baths, followed by chromium plating wastewater and pre-cleaning wastewater. As a PFOS substitute, OBS has been confirmed to exhibit similar toxicity and is used in firefighting foams, oil recovery additives, paper wetting agents, ink additives, and electroplating cleaners [36]. Its frequent detection in electroplating systems indicates its widespread use, warranting future attention. Non-target screening of commercial chromium mist suppressants further identified hydrogen-substituted perfluoroalkane sulfonates (H-PFSAs) and perfluoroether sulfonates (O-PFSAs) and discovered for the first time that 8:2 Cl-PFAES, a long-chain chlorinated ether sulfonate, has been practically applied as a PFOS substitute, while some labeled fluorine-free products are actually hydrocarbon surfactants [28].

In addition to structural identification, recent efforts in non-targeted analysis have focused on the semi-quantification of newly identified PFASs to support preliminary risk assessment in the absence of authentic reference standards. Current strategies typically employ surrogate-based approaches, in which the response of an unknown PFAS is estimated against a structurally similar standard. To improve accuracy, various surrogate selection criteria have been explored, including chemical fingerprints and fluorine content [37,38]. Additionally, the integration of non-targeted screening data with total organofluorine measurements, such as the Total Oxidizable Precursor (TOP) assay, Extractable Organic Fluorine (EOF), and Adsorbable Organic Fluorine (AOF), provides a mass balance approach to evaluate the completeness of PFAS identification and the significance of the unknown fraction [39,40]. This combined workflow, from discovery to semi-quantitative assessment, is essential for a comprehensive understanding of PFAS contamination in complex matrices such as electroplating wastewater.

## 4. Treatment Technologies for PFASs in Electroplating Wastewater

Electroplating wastewater contains high concentrations of PFASs, heavy metals, acids/bases, oils, and complexing agents, posing challenges for PFAS removal. Treatment typically involves multi-stage combinations including coagulation/flocculation, sedimentation, flotation, biological treatment, adsorption, membrane separation, electro-oxidation, and hybrid treatment trains. This section systematically reviews the performance, mechanisms, influencing factors, and engineering limitations of each technology based on field monitoring and literature data.

### 4.1. Coagulation/Flocculation

Coagulation/flocculation is typically employed as a pretreatment step in wastewater treatment. In a typical coagulation/flocculation process, a coagulant (e.g., aluminum or iron salts) is added to wastewater under rapid mixing to neutralize charges and destabilize suspended particles, followed by slow mixing to promote floc formation. The flocs are then removed by sedimentation or flotation. However, this process is designed for suspended solids and heavy metals, not for dissolved PFASs.

PFASs contain strong ionic hydrophilic groups (sulfonate, carboxylate), making them highly water-soluble and resistant to sedimentation. In neutral to weakly alkaline (pH 7–10) conditions common in chromium plating wastewater, both chromium hydroxide flocs and PFAS anions carry negative charges, causing electrostatic repulsion and poor PFAS removal, sometimes even negative. For example, Jiang et al. [19] reported PFOS removal of −55% to 7% and 6:2 FTS removal of 0% to 21% after coagulation/flocculation, attributed to precursor transformation and adsorption–desorption imbalances.

PFAS behavior during coagulation is pH-dependent. At pH 7–10, hydrophobic PFOS tends to desorb from suspended particles due to electrostatic repulsion, increasing aqueous concentration [41,42]; less hydrophobic 6:2 FTS remains dissolved and shows stable concentrations [43,44]. Under strongly alkaline conditions (pH > 12), short-chain PFCAs (C4–C7) can form insoluble salts with Ca^2+^, Mg^2+^, Fe^3+^, and Al^3+^, with removal decreasing as chain length increases; PFOA removal becomes negative due to strong desorption [41,42]; PFSAs cannot form insoluble metal salts and exhibit negative removal due to increased desorption [32,41,42,45,46,47]. Liu et al. [32] reported that under strongly alkaline coagulation, removal rates for PFCAs (C5–C8) were 91%, 26%, 21%, and −31%; for PFSAs (C4, C6, C8, C10), removal rates were −18%, −160%, −470%, and −2800%.

### 4.2. Flotation

In a typical flotation process, fine gas bubbles (usually air) are introduced into the wastewater, either by dissolved air flotation or mechanical aeration. As the bubbles rise through the water column, surface-active compounds such as PFASs adsorb onto the bubble surfaces due to their amphiphilic nature. The bubble-PFAS aggregates then rise to the surface, forming a foam layer that is continuously or periodically skimmed off for collection and further treatment, while the clarified water is discharged from the bottom.

Flotation is typically placed after coagulation/flocculation and before sedimentation, or as a substitute for sedimentation. Its PFAS removal effectiveness depends on floc retention, process sequence, and PFAS functional groups. Flotation relies on microbubbles for solid–liquid separation via air-liquid interface enrichment, floc co-flotation, and ion bridging. PFASs, being strong surfactants, accumulate at bubble surfaces; metal hydroxide flocs adsorb PFASs via hydrophobic interactions, electrostatic attraction, or ion bridging; cations like Ca^2+^, Mg^2+^, Fe^3+^, and Al^3+^ can act as ion bridges, enhancing PFAS removal.

Process sequence is critical. Direct flotation after flocculation retains high floc concentrations, maximizing adsorption-flotation synergy, and achieving effective PFSA removal. In contrast, flotation after sedimentation loses flocs, leading to poor PFCAs removal and possible desorption-induced concentration increases. Jiang et al. [19] reported that direct flotation after flocculation achieved 88% PFOS removal. Liu et al. [32] found that in a coagulation-sedimentation-flotation system, PFOS removal in the flotation unit was only 8%.

Functional groups also matter. PFSAs are more hydrophobic and surface-active, favoring removal; PFCAs are more polar and hydrophilic, leading to poor or negative removal. Liu et al. [32] reported that PFCA (C5–C8) removal ranged from −77% to −15%, while PFSA removal ranged from 8% to 35%. Thus, flotation is not a universal PFAS removal technology; its effectiveness is constrained by floc retention, functional group selectivity, and process integration.

### 4.3. Biological Treatment

In a typical biological treatment process, wastewater is fed into a bioreactor (e.g., activated sludge tank or biofilm reactor), where microorganisms degrade organic matter under aerobic or anaerobic conditions over a certain hydraulic retention time. The treated effluent is then separated from the biomass by sedimentation, and part of the biomass is returned to the reactor to maintain microbial activity.

Biological treatment (anaerobic/aerobic digestion, biofilters) removes conventional pollutants via microbial metabolism but has almost no degradation capability for PFASs [32,33]. A recent meta-analysis of 97 microbial PFAS biotransformation studies confirmed that the likelihood of PFAS biotransformation is higher under aerobic conditions, in defined or axenic cultures, and when PFASs contain fewer fluorine atoms in the molecule, yet the literature remains scarce in analyses of biotransformation products and identification of responsible microorganisms and enzymes [48]. Furthermore, a systematic review concluded that no enzyme has been experimentally validated to catalyze efficient cleavage of perfluorinated C–F bonds under environmentally relevant conditions, and most reported biotransformations involve precursor compounds rather than terminal PFASs [49].

Microbial communities in electroplating wastewater systems are dominated by heterotrophs that degrade C–H, C–C, C–O, ester, and amide bonds but cannot cleave C–F bonds (bond energy ~485 kJ/mol) and lack PFAS-degrading enzymes. Autotrophs (e.g., nitrifiers) play no role in PFAS degradation. While specific bacterial strains such as *Pseudomonas* spp., *Acidimicrobium* sp. strain A6, and *Labrys portucalensis* F11 have demonstrated PFAS transformation under laboratory conditions [50], these findings are primarily from pure culture studies with high PFAS concentrations and have not been validated in full-scale WWTP biological treatment systems [48,51].

Biological treatment not only fails to degrade PFASs but can also promote precursor biotransformation, increasing aqueous PFAS concentrations. Research on aerobic wastewater microbial consortia demonstrated that PFOA, PFOS, and 6:2 FTS were transformed (not mineralized) by 47.87%, 28.96%, and 43.1%, respectively, with the generation of shorter-chain compounds (carboxylates and sulfonates) and only limited defluorination [52]. This indicates structural modification rather than effective destruction of PFASs. The microbial genera *Methylophilus*, *Acidomonas*, *Pseudomonas*, *Clostridium*, *Klebsiella*, and *Acinetobacter* were positively correlated with PFAS degradation in aerobic cultures [52]. Wu et al. [33] reported that after biological treatment in a Jiangsu electroplating plant, PFOA, PFOS, and PFBA concentrations increased by 50–80%. Electroplating wastewater contains fluorotelomer alcohols and sulfonamide precursors; under aerobic conditions, heterotrophs can convert precursors like 6:2 fluorotelomer sulfonamide alkylbetaine (6:2 FTAB), 6:2 fluorotelomer sulfonamide propyl N,N-dimethylamine (M4), N-ethyl perfluorooctane sulfonamide (N-EtFOSA), and N-methyl perfluorooctane sulfonamide (N-MeFOSA) via hydrolysis, oxidation, decarboxylation, and hydroxylation to form stable PFASs (PFBA, PFOA, PFOS) [53]. Geng and Helbling (2026) identified 71 biotransformation products from perfluoroalkane sulfonamide derivatives in aerobic batch experiments with wastewater microbial communities, demonstrating the formation of key intermediates during precursor transformation [54]. Similar phenomena are observed in textile and municipal wastewater [21,55]. Zhang et al. (2026) reported that in a sequencing batch biofilm reactor, biotransformation accounted for 13.8% removal of PFHxA and 14.9% removal of GenX, with upregulation of *CrcB/F1A* genes in *Pseudomonadaceae* suggesting microbial degradation potential; however, these removal rates may also include adsorption to biomass [56]. Therefore, biological treatment is ineffective for PFAS removal and may exacerbate PFAS pollution.

### 4.4. Adsorption

In a typical adsorption process, wastewater is passed through a packed-bed column or contacted with adsorbent material in a batch or continuous-flow configuration. As the water flows through the adsorbent bed, PFAS molecules are retained on the adsorbent surface through various mechanisms (e.g., hydrophobic interactions, electrostatic attraction). The treated effluent exits the column, and once the adsorbent reaches breakthrough, it is removed for regeneration or disposal and replaced with fresh adsorbent.

Adsorption is the most mature and widely applied PFAS removal technology, typically as a polishing step. Granular activated carbon (GAC) and powdered activated carbon (PAC) are common adsorbents. Activated carbon removes PFASs primarily through hydrophobic interactions between the non-polar fluorinated chain and the hydrophobic carbon surface via van der Waals forces. This mechanism favors long-chain PFASs (e.g., PFOS, PFOA) over short-chain ones, as longer fluorinated chains provide stronger hydrophobic interactions. However, they are less effective for short-chain and some emerging PFASs [57]. For example, GAC achieved 60–99% removal of long-chain PFOS but only 40–70% removal of 6:2 FTS, perfluorobutanoic acid (PFBA), and perfluorohexanoic acid (PFHxA) [19]. Co-occurring organics and surfactants compete for adsorption sites, accelerating GAC breakthrough [58,59]. GAC is low-cost and mature but suffers from limited short-chain removal, frequent replacement, and secondary pollution from incineration [60,61].

Ion exchange resins outperform activated carbon, particularly strong-base anion exchange resins containing quaternary ammonium groups (R_4_N^+^) as positively charged sites. The negatively charged head groups of PFASs, including sulfonate (R–SO_3_^−^) and carboxylate (R–COO^−^), are electrostatically attracted to these sites, forming ion pairs. Long-chain PFASs benefit from additional hydrophobic interactions between their fluorinated tails and the resin matrix, while short-chain PFASs exhibit weaker binding due to lower hydrophobicity and higher solubility, leading to earlier breakthrough. Competing anions (e.g., sulfate, chloride) can also occupy exchange sites, reducing PFAS capacity. They achieve higher capacity and more stable removal, lowering effluent PFASs to ng/L levels. In a Jiangsu park study, anion exchange resin (AER) achieved 98% and 85% removal for PFOS and 6:2 FTS, respectively, with a breakthrough volume of 3600 BV [22]. AER nearly completely removed PFOS, PFHpS, and PFHxS (>98%, no breakthrough), but 6:2 FTS broke through quickly, with effluent concentrations averaging 0.5 μg/L [22]. However, ion exchange resins are difficult to regenerate and are often single-use [62,63,64].

Recent adsorbent development focuses on multi-mechanism synergy (e.g., amine-functionalized mesoporous silica, COFs, MOFs) to combine electrostatic and hydrophobic interactions. However, most are only batch-tested; column applications lack maturity. Modified clay adsorbents are commercialized, low-cost, organic-resistant, and PFAS-selective [65], but pilot-scale behavior remains unclear [66,67].

To address the limitations of adsorbent disposal, various regeneration and detoxification techniques have been developed. For activated carbon, thermal regeneration (600–1000 °C) achieves > 90% desorption efficiency but is energy-intensive and may release volatile organofluorine compounds [68,69]. Hydrothermal alkaline treatment (120–240 °C) offers a low-temperature alternative, achieving up to 97% defluorination of adsorbed PFOA while restoring GAC capacity over multiple cycles [70]. Emerging methods include supercritical CO_2_ extraction and electrochemical in situ regeneration [23]. For ion exchange resins, regeneration typically requires salt solutions (e.g., NaCl, NH_4_Cl) combined with organic solvents (e.g., methanol, ethanol), as single-component regenerants are insufficient [62,71]. The resulting PFAS-laden regenerant stream can be further treated by UV/sulfite reduction, which generates hydrated electrons (e^−^aq) to degrade PFASs (>99%) and achieve defluorination (~90%) [72,73,74]. In all cases, the PFAS-containing stream requires additional treatment to achieve complete mineralization and prevent secondary pollution [61].

### 4.5. Membrane Separation

Membrane separation, including ultrafiltration (UF), nanofiltration (NF), and reverse osmosis (RO), is typically used as a polishing step. In this process, wastewater is pressurized through a membrane module; water passes through as permeate, while PFASs and other solutes are retained as concentrate. Removal occurs via size exclusion, electrostatic repulsion, and hydrophobic adsorption.

In real electroplating wastewater, long-chain PFAS removal is high, but short-chain and emerging PFAS removal is limited. Qu et al. [20] reported that UF/RO removed ~85.5% of PFOS (5500 μg/L → 795 μg/L) but only ~44.7% of 6:2 Cl-PFESA (154.5 μg/L → 85.4 μg/L). Wu et al. [33] found that RO-containing trains achieved 93–99% total PFAS removal, while membrane-free trains showed increased PFAS concentrations (12.4%). Long-chain PFASs (PFOS, PFHxS, PFHpS) are almost completely removed, but short-chain PFASs (PFBA, PFHxA, 6:2 FTS) exhibit lower removal due to small molecular size and high solubility. High salinity in real wastewater causes electrostatic shielding, reducing membrane-PFAS repulsion and promoting short-chain breakthrough. Co-existing organics and heavy metals compete for sites and worsen fouling; pH (2–12) and temperature (40–60 °C) affect surface charge and PFAS diffusivity [75,76]. Thus, laboratory high removal rates may not translate to field applications [77,78].

Membrane separation generates concentrated retentate that requires further treatment or disposal, which remains a significant challenge for its practical application in electroplating wastewater treatment.

### 4.6. Electro-Oxidation

In a typical electro-oxidation process, wastewater is placed in an electrochemical cell equipped with an anode and a cathode. A direct current is applied, generating reactive oxygen species (e.g., hydroxyl radicals) at the anode surface through water electrolysis. These species attack PFAS molecules, breaking the C–F bonds and leading to their mineralization or transformation into shorter-chain intermediates. Treated water is then discharged, and electrodes require periodic cleaning to remove scaling or fouling.

Electro-oxidation is an emerging destruction technology for in situ water treatment that generally does not require continuous chemical addition [79,80]. It oxidizes compounds via direct electron transfer or hydroxyl radicals (•OH) generated from water electrolysis. In anodic oxidation, PFAS molecules lose electrons to the anode surface, initiating decarboxylation of the carboxylate head group (–COO^−^) or desulfonation of the sulfonate head group (-SO_3_^−^), followed by attack of •OH on the weakened C–F bonds. The high C–F bond energy (~485 kJ/mol) makes PFASs highly resistant to oxidation, requiring high-potential electrode materials such as boron-doped diamond (BDD) [79,80]. Based on the degradation pathways reported for different PFAS classes, the decomposition mechanisms vary depending on the head group and chain length.

The degradation of PFCAs proceeds through Kolbe decarboxylation to form perfluoroalkyl radicals, followed by stepwise CF_2_ elimination to generate shorter-chain PFCAs [81,82,83,84,85]. For PFSAs (e.g., PFOS), cleavage of the C–S bond (bond energy ~272 kJ/mol) is more favorable than C–C bond cleavage (~346 kJ/mol), followed by similar stepwise decomposition [86]. Degradation rates are structure-dependent: longer-chain PFASs degrade faster than shorter-chain homologs, and PFCAs degrade faster than PFSAs of the same chain length due to the lower bond dissociation energy of C–F bonds adjacent to the carboxylate group [87,88,89].

Despite its effectiveness, the application of electro-oxidation to real electroplating wastewater faces several challenges. Firstly, the high energy consumption and relatively low treatment capacity limit its use to small-volume, high-concentration streams [90]. Secondly, the presence of co-existing organics and chloride ions in electroplating wastewater can compete for reactive species and generate toxic byproducts such as chlorate and perchlorate [90]. Additionally, while electro-oxidation has shown promising results in lab-scale studies, achieving over 75% PFAS removal in leachate after 8 h [91], its performance in complex electroplating matrices requires further validation. Several studies have explored its application in electroplating wastewater. Su et al. reported that a titanium-supported nano-silver electrode achieved 92.7% removal of PFOA and 94.0% removal of PFOS at pH 7 and 25 mA/cm^2^ within 60 min [92]. A sequential electrocoagulation-electrooxidation process using a Ti/TiO_2_-RuO_2_-SnO_2_ anode removed over 99% COD and 92% TOC from real Teflon plating wastewater [93]. Pilot-scale studies using Magnéli phase Ti_4_O_7_ anodes demonstrated effective degradation of eight perfluoroalkyl acids (PFAAs), with performance dependent on electrolyte type and current density. Furthermore, EO can reduce hexavalent chromium (Cr^6+^) to trivalent chromium (Cr^3+^), offering additional benefits for electroplating wastewater treatment.

Other electrochemical advanced oxidation processes, such as electro-Fenton, have also been explored for PFAS degradation; however, they require Fe^2+^ addition and remain less studied for real electroplating wastewater compared to anodic oxidation. For electro-oxidation itself, further research is needed to improve energy efficiency, reduce electrode costs, and suppress toxic byproduct formation [94].

To provide a clear and systematic overview of the individual treatment technologies discussed in Section 4.1, Section 4.2, Section 4.3, Section 4.4, Section 4.5 and Section 4.6, their key performance parameters, including materials/chemicals, operating conditions, target PFASs, removal efficiency, advantages, disadvantages, and environmental impacts, are summarized in Table 1 and Table 2.

### 4.7. Hybrid Treatment Trains

To overcome the limitations of individual technologies, various hybrid treatment trains combining separation and destruction processes have been explored. For instance, nanofiltration followed by electrochemical oxidation effectively concentrates PFASs and subsequently degrades the concentrate, with over 96% PFAS removal and 98% degradation of PFHxA reported [95]. Other innovative combinations include membrane distillation with electro-Fenton and anodic oxidation (100% PFOA removal, ~95% TOC mineralization) [96], surfactant-assisted ultrafiltration (PFOA rejection increased from 30.3% to 99.1%) [97], integrated membrane capacitive deionization (68.66% removal) [98], and MXene-TiO_2_ enhanced RO membranes (96.9% PFBA, 98.8% PFOA removal) [99]. Foam fractionation coupled with electro-oxidation has also been applied, achieving over 80% removal of long-chain PFASs and 30% removal of short-chain PFASs [14].

Despite these promising results, most hybrid processes remain at the laboratory or pilot scale and require further validation in real electroplating wastewater. Key challenges include process optimization, byproduct management, and economic feasibility. Future research should focus on pilot-scale demonstrations and life-cycle cost analysis.

## 5. Conclusions

PFASs in electroplating wastewater mainly originate from chromium mist suppressants used in chromium plating. Long-chain C8 compounds, including PFOS, 6:2 FTS, and 6:2 Cl-PFESA, dominate, with concentrations up to 3.6 × 10^7^ ng/L. Temporal data from 2013 to 2025 reveal a clear substitution trajectory. PFOS dominated before 2020, 6:2 Cl-PFESA emerged during 2020–2022, and 6:2 FTS has become dominant since 2022. Non-target screening reveals widespread hidden PFASs such as H-PFBA and OBS, accounting for 13% to 87% of total PFASs across different wastewater streams, indicating that target analysis alone significantly underestimates actual pollution levels.

Conventional technologies, including coagulation, flotation, and biological treatment, show limited PFAS removal and may even increase aqueous PFAS concentrations due to precursor transformation. Established treatment technologies such as adsorption and membrane separation, as well as emerging destructive technologies like electro-oxidation, are effective for long-chain PFASs. For short-chain and emerging PFASs, however, their performance is comparatively limited. Electro-oxidation requires higher energy input and longer treatment times to achieve comparable degradation, while adsorption and membrane separation suffer from weaker retention. Engineering challenges, including adsorbent regeneration, membrane fouling, concentrate disposal, and toxic byproduct formation such as chlorate and perchlorate, remain to be addressed.

Addressing these challenges requires coordinated efforts on both analytical characterization and treatment technology development. On the characterization side, a reliable workflow from identification to quantification is essential for accurately profiling PFASs in complex matrices such as electroplating wastewater. Future monitoring should integrate target analysis, non-target screening, and total organofluorine measurements such as EOF, AOF, and TOP assays to establish baseline fingerprints for different electroplating processes, enabling early detection of emerging substitutes before they become widespread. On the treatment side, given the limitations of individual technologies, hybrid treatment trains that combine separation processes (such as adsorption or membrane filtration) with destructive processes, such as electro-oxidation or advanced reduction, should be developed, with pilot-scale demonstrations in representative electroplating parks to evaluate performance, energy consumption, and byproduct formation under real-world conditions. Life-cycle cost and byproduct management assessments are also essential to guide practical implementation.

## Figures and Tables

**Table 1:** Comparison of PFAS treatment technologies for electroplating wastewater.

Treatment Technology	Materials/Chemicals	Operating Conditions	Target PFASs	Removal Efficiency	Ref.
Coagulation /Flocculation	Alum (aluminum sulfate), ferric chloride, polyaluminum chloride, PAM	pH 7–10; rapid mixing (coagulation) followed by slow mixing (flocculation); sedimentation for solid–liquid separation	PFOS, 6:2 FTS	PFOS: −55% to 7%; 6:2 FTS: 0% to 21%	[19]
Flotation	Microbubbles (air), surfactants (optional, e.g., cationic or zwitterionic surfactants)	Direct flotation after coagulation (optimal); flotation after sedimentation (ineffective); bubble generation via dissolved air flotation or mechanical aeration	PFSAs (PFOS, PFHxS), PFCAs (PFOA, PFHxA)	PFSAs: 8–88%; PFCAs: −77% to −15%	[19,32]
Biological treatment	Activated sludge, biofilm carriers (e.g., biofilter, MBBR), aerobic/anaerobic microorganisms	HRT (hydraulic retention time); aerobic (with aeration) or anaerobic (sealed and mixed) conditions; pH 6.5–8.5; biomass recycling via sedimentation	PFOA, PFOS, PFBA	Negative removal (PFAS concentrations increase by 50–80% after treatment)	[33]
Activated carbon adsorption	GAC (granular activated carbon), PAC (powdered activated carbon)	Packed-bed column; EBCT (empty bed contact time); flow rate; regeneration required after breakthrough	PFOS, PFBA, PFHxA, 6:2 FTS	PFOS (long-chain): 60–99%; short-chain (PFBA, PFHxA, 6:2 FTS): 40–70%	[19,22,45]
Ion-Exchange Resins	Strong-base anion exchange resins (AER) with quaternary ammonium functional groups (R_4_N^+^), brine for regeneration (limited)	Packed-bed column; flow rate; BV (bed volume); short EBCT; single-use (difficult to regenerate)	PFOS, 6:2 FTS, PFHxS, PFHpS	PFOS: >98% (no breakthrough); 6:2 FTS: 85% (breakthrough at 3600 BV); PFHxS and PFHpS: >98%	[22]
Membrane separation	UF (ultrafiltration), NF (nanofiltration), RO (reverse osmosis) membranes; polyamide or other semi-permeable materials	Applied pressure (UF: low; NF: medium; RO: high); temperature (40–60 °C); pH (2–12); cross-flow velocity; periodic backwashing and chemical cleaning	PFOS, 6:2 Cl-PFESA, PFBA, PFHxA, 6:2 FTS	Long-chain (PFOS, PFHxS, PFHpS): >99% (near complete); short-chain (PFBA, PFHxA, 6:2 FTS): <50%	[20,33]
Electro-oxidation	BDD (boron-doped diamond) electrodes, Ti_4_O_7_ (Magnéli phase) electrodes, nano-silver electrodes (Ti-supported), PbO_2_, SnO_2_ electrodes	Current density (e.g., 25 mA/cm^2^); pH (e.g., pH 7); reaction time (e.g., 60 min); direct current applied; periodic electrode cleaning to remove scaling/fouling	PFOA, PFOS, short-chain PFASs	PFOA: 92.7%; PFOS: 94.0% (nano-Ag electrode, 60 min, 25 mA/cm^2^ pH 7)	[92]

**Table 2:** Advantages and disadvantages of the technologies used for the treatment of PFASs from electroplating wastewater.

Treatment Technology	Advantages	Disadvantages	Environmental Impacts
Coagulation /Flocculation	Simple operation; low capital cost; widely applied for conventional pollutants (suspended solids and heavy metals)	Very poor PFAS removal (even negative removal); pH-dependent; affected by coexisting ions; generates large volumes of chemical sludge	Chemical sludge requiring disposal; chemical additive input; potential secondary pollution
Flotation	Effective for PFSAs (up to 88%); utilizes PFAS surface activity; no chemical addition (unless boosting agents are used)	Poor removal for PFCAs; process sequence dependent (efficiency decreases significantly if preceded by sedimentation); limited for short-chain PFASs	PFAS-laden foam requiring further treatment; moderate energy consumption for bubble generation
Biological treatment	Effective for conventional organics and nutrients; low operating cost; simple operation	Cannot degrade PFASs (C–F bonds not cleaved); may increase dissolved PFAS concentrations via precursor transformation; no PFAS removal capability	Low direct energy input; biosolids may contain PFASs; increased downstream treatment burden
Activated carbon adsorption	Excellent for long-chain PFASs (60–99% removal); mature and well-established technology; simple operation; regenerable	Poor for short-chain PFAS; affected by competing organics (TOC fouling); short bed life; frequent replacement; high operating cost	Spent GAC requires thermal regeneration or incineration; potential incomplete PFAS destruction and release of fluorinated byproducts; high energy consumption
Ion-Exchange Resins	High PFOS removal (up to 98%); stable performance for long-chain PFASs; low empty bed contact time (EBCT); small footprint; effluent at ng/L levels	Short-chain PFAS breakthrough; difficult to regenerate (often single-use); affected by competing anions (e.g., sulfate); high cost	Spent resin disposal challenges; incineration may release harmful gases; chemical inputs for resin production and use
Membrane separation	Near 100% removal for long-chain PFASs; broad-spectrum pollutant rejection; high effluent quality; applicable to various water matrices	Membrane fouling; concentrate disposal; high energy consumption; limited removal for short-chain PFASs; high capital and maintenance costs	PFAS-laden concentrate requiring further treatment; membrane cleaning chemicals; membrane waste disposal
Electro-oxidation	Can mineralize PFASs (C–F bond cleavage); no chemical addition; in situ treatment; effective for small volumes of high-concentration wastewater	High energy consumption; expensive electrode materials (e.g., BDD); affected by coexisting organics; potential formation of toxic byproducts (e.g., chlorate, perchlorate); long treatment time	High carbon footprint; potential toxic byproducts; electrode materials may involve rare metals; suitable as a polishing step for concentrate

## Data Availability

No new data were created or analyzed in this study. Data sharing is not applicable to this article.

## Abbreviations

PFASs	Per- and polyfluoroalkyl substances
PFCAs	Perfluorocarboxylic acids
PFSAs	Perfluoroalkane sulfonic acids
PFOS	Perfluorooctane sulfonic acid
PFOA	Perfluorooctanoic acid
PFHxS	Perfluorohexane sulfonic acid
PFHpS	Perfluoroheptane sulfonic acid
PFBA	Perfluorobutanoic acid
PFHxA	Perfluorohexanoic acid
PFPeA	Perfluoropentanoic acid
PFDA	Perfluorodecanoic acid
PFNA	Perfluorononanoic acid
PFUnDA	Perfluoroundecanoic acid
PFDoA	Perfluorododecanoic acid
PFTrDA	Perfluorotridecanoic acid
PFTeDA	Perfluorotetradecanoic acid
PFHxDA	Perfluorohexadecanoic acid
PFODA	Perfluorooctadecanoic acid
PFBS	Perfluorobutane sulfonic acid
PFPeS	Perfluoropentane sulfonic acid
PFDS	Perfluorodecane sulfonic acid
6:2 Cl-PFESA	6:2 Chlorinated polyfluoroalkyl ether
8:2 Cl-PFESA	8:2 Chlorinated polyfluoroalkyl ether
4:2 FTS	4:2 Fluorotelomer sulfonate
6:2 FTS	6:2 Fluorotelomer sulfonate
8:2 FTS	8:2 Fluorotelomer sulfonate
OBS	Sodium *p*-perfluorous nonenoxybenzenesulfonate
HFPO-DA	Hexafluoropropylene oxide dimer acid
HFPO-TA	Hexafluoropropylene oxide timer acid
9Cl-PF3NS	9-Chloroperfluoro-3-oxanonane
6:2 FTCA	6:2 Fluorotelomer carboxylic acid
PFECHS	Perfluoroethylcyclohexane sulfonic acid
H-PFBA	Monohydro-substituted perfluorobutanoic acid
H-PFCAs	Hydrogen-substituted perfluoroalkyl carboxylic acids
H-PFSAs	Hydrogen-substituted perfluoroalkane
O-PFSAs	Perfluoroether sulfonates
6:2 FTAB	6:2 Fluorotelomer sulfonamide alkylbetaine
M4	6:2 Fluorotelomer sulfonamide propyl N,N-dimethylamine
N-EtFOSA	N-ethyl perfluorooctane sulfonamide
N-MeFOSA	N-methyl perfluorooctane sulfonamide
EOF	Extractable Organic Fluorine
AOF	Adsorbable Organic Fluorine
TOP	Total Oxidizable Precursor
